# Ionic Liquids: Efficient Media for the Lipase-Catalyzed Michael Addition

**DOI:** 10.3390/molecules23092154

**Published:** 2018-08-27

**Authors:** Yunchang Fan, Dongxu Cai, Xin Wang, Lei Yang

**Affiliations:** College of Chemistry and Chemical Engineering, Henan Polytechnic University, Jiaozuo 454003, China; CDX940407@163.com (D.C.); asnol21@163.com (X.W.); xcy78413@tom.com (L.Y.)

**Keywords:** ionic liquids (ILs), lipase, Michael addition, warfarin, bio-catalysis

## Abstract

Recently, ionic liquids (ILs) have been regarded as ideal media for non-aqueous bio-catalysis. In this work, the synthesis of warfarin by the lipase-catalyzed Michael addition in IL media and the parameters that affected the warfarin yield were investigated. Experimental results demonstrated that the chemical structures of the ILs were a major factor for influencing the warfarin yield. The ILs containing the NTf_2_^–^ anion were suitable reaction media due to the high chemical stability of this anion. The incorporation of the hydroxyl group on the IL cation significantly improved the lipase activity due to the H_2_O-mimicking property of this group. The lipase activity decreased by increasing the alkyl chain length on the IL cation due to the non-polar domain formation of the IL cation at the active site entrance of lipase. The ILs and lipase could be reused no less than five times without reduction in the warfarin yield.

## 1. Introduction

In recent decades, enzymes have been regarded as green catalysts because they are recyclable non-toxic materials and the enzymatic reactions are usually performed under mild conditions [1,2]. Lipases are the most used enzymes because of their high stability, activity, wide range of substrates, and low cost [3,4,5,6]. They can catalyze many reactions such as the Michael addition [3,4,5], esterification [6], ester-hydrolysis [7], and transesterification [8]. Among these reactions, Michael addition is one of the most effective ways to form mild C–C bonds and generate interesting, drug-like scaffolds. The synthesis of warfarin by Michael addition of 4-hydroxycoumarin (4-HC) to benzylideneacetone (BA) is a classic case. Warfarin and sodium warfarin are widely used as vitamin K antagonists, which prevent the vitamin K-dependent synthesis of blood-clotting proteins and, thus, decrease blood coagulation [9,10]. Xie et al. [11] reported the synthesis of warfarin and derivatives via the lipase-catalyzed Michael addition in dimethyl sulfoxide (DMSO) media, which presented a yield of 87%. Sano et al. [5] also reported the lipase-catalyzed Michael reaction of 4-HC to BA in anhydrous DMSO and a higher yield (85%) was achieved. Although some interesting results have been reported by these excellent papers, there is still one problem [3,5,11]. Although lipases exhibit the highest activity in DMSO medium, DMSO is flammable and toxic even at low concentrations (<10%, *v*/*v*) [12]. Therefore, it is of great importance to find environmentally-benign solvents. In this context, ionic liquids (ILs) are considered as environmentally-benign solvents compared to the conventional solvents because of their negligible vapor pressure, high dissolving power, and high thermal stability and recyclability [13,14,15]. At present, ILs have found applications in diverse areas such as the conversion of biomass [15] and biocatalysis [16,17,18,19]. Schutt et al. studied the impact of water-dilution on the solvation properties of an ester-functionalized IL for model biomass molecules through the use of biased and unbiased molecular dynamics (MD) simulations. It is found that hydrogen-bonding interactions between the IL anion and water are a major driving force that significantly impacts the solvent properties of the ester-functionalized IL as well as conformational preferences of the cellulosic model compound [15]. Burney et al. studied the impact of imidazolium-based ILs on enzyme functionality by using molecular simulations. It is found that the enzymes lipase and α-chymotrypsin are altered by randomly mutating lysine surface residues to glutamate in aqueous ILs 1-butyl-3-methylimidazolium chloride and 1-ethyl-3-methylimidazolium ethyl sulfate. These mutations resemble succinylation of the enzyme by chemical modification, which enhances the stability of both enzymes in ILs [19]. Very recently, the lipase-catalyzed esterification [20], ester-hydrolysis [21], and transesterification [22] reactions in the IL media have been widely reported. Furthermore, many reports have demonstrated that the ILs with the anions PF_6_^–^ and BF_4_^–^ were suitable reaction media for the lipase-catalyzed transesterification [22,23] and esterification [24,25] reactions. However, the anions PF_6_^–^ and BF_4_^–^ are hydrolytically unstable, which results in a propensity to decompose and release corrosive HF [26,27]. It has been proven that bis(trifluoromethanesulphonyl)imide (NTf_2_^–^) has the higher chemical stability than PF_6_^–^ and BF_4_^–^ [27,28]. Some work suggests that the hydroxyl-functionalized ILs have a good compatibility with biomolecules such as catalase [29] and cytochrome c [30]. Therefore, it will be expected that the hydroxyl-functionalized ILs containing NTf_2_^–^ can be used as ideal media for the lipase-catalyzed Michael addition reaction.

In this paper, the feasibility of the use of the hydroxyl-functionalized ILs as the reaction media of lipase-catalyzed Michael addition and the parameters affecting the reaction yield have been investigated systematically. Furthermore, the recyclability of ILs and lipase has also been studied. For clarity, the names and abbreviations of the ILs are listed in Table 1.

## 2. Results and Discussion

### 2.1. Selection of ILs

**Figure 1:**
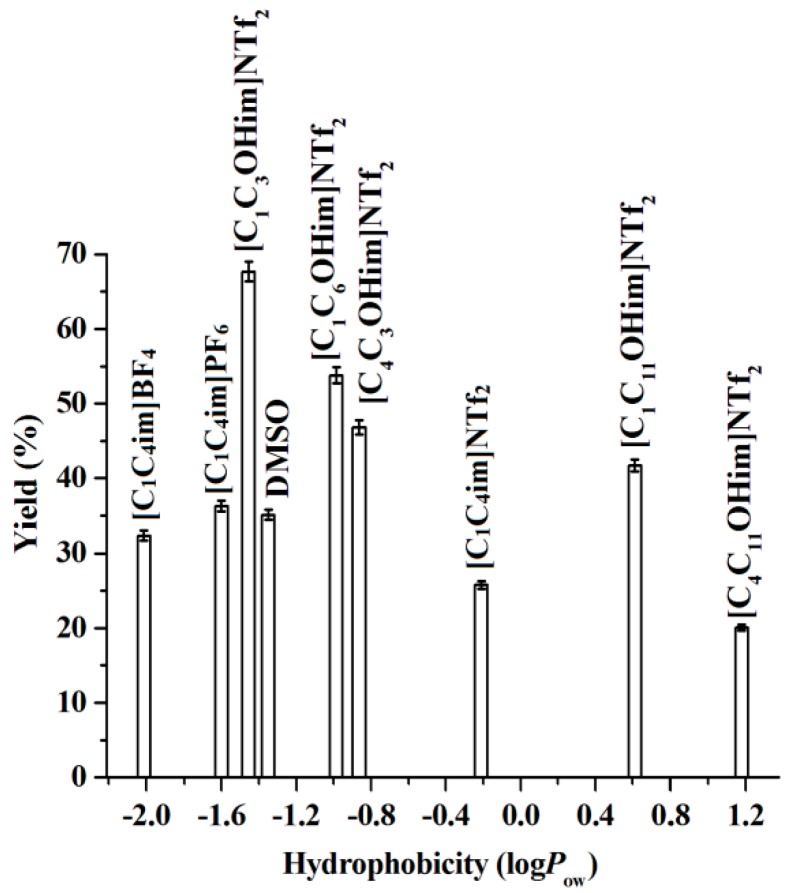
The correlation of the hydrophobicity of solvents used in this work and the warfarin yield. Reaction conditions: BA, 1.0 mmol, water, 0.1 mL, reaction time, 96 h, temperature, 45 °C, lipase, 30 mg, 4-HC, 0.5 mmol, [C_1_C_3_OHim]NTf_2_, 2.0 g.

In this work, eight kinds of ILs and DMSO have been investigated systematically in order to find the most suitable reaction medium. As shown in Figure 1, the lipase activity in dialkylimidazolium-based IL media follows the order: [C_1_C_4_im]PF_6_ > [C_1_C_4_im]BF_4_ > [C_1_C_4_im]NTf_2_. It has been reported that the nucleophilicity of anions increases in the following trend: PF_6_^–^ < BF_4_^–^ < NTf_2_^–^ where the more nucleophilic anions have a tendency to interact with the positively charged sites in the enzyme structure and to change the enzyme’s conformation, which could eventually result in a denaturation of the enzyme [31,32,33]. However, PF_6_^–^ and BF_4_^–^ are moisture-sensitive anions and tend to hydrolyze and release toxic HF. From the point of view of safety and environmental protection, the use of NTf_2_^–^ is more preferable [26,27].

An interesting phenomenon, seen in Figure 1, is that the incorporation of the hydroxyl group on the IL cation significantly improves the enzyme activity ([C_1_C_3_OHim]NTf_2_ versus [C_1_C_4_im]NTf_2_) and the lipase activity in [C_1_C_3_OHim]NTf_2_ is also much higher than in DMSO. This accounts for the fact that the essential water layer around the lipase molecule maintains the enzyme activity in organic media. The IL [C_1_C_3_OHim]NTf_2_ contains a hydroxyl group and holds the H_2_O-mimicking property and hydrogen-bonding functionality, which helps the enzyme to maintain its flexible and active conformation [30,34,35].

Lastly, as shown in Figure 1, the lipase activity in the hydroxyl-functionalized ILs follows the order: [C_1_C_3_OHim]NTf_2_ > [C_1_C_6_OHim]NTf_2_ > [C_4_C_3_OHim]NTf_2_ > [C_1_C_11_OHim]NTf_2_ > [C_4_C_11_OHim]NTf_2_, which is opposite to the order of their hydrophobicity. It is known that the entrance of active sites of lipase is formed by the non-polar residues. The IL cation can diffuse into the active sites of lipase driven by van der Waals interactions. This means that the substrates have to compete with the IL cations to reach the active sites [33,36]. The increase in the hydrophobicity results in the increase in the van der Waals interactions between the alkyl chains of the ILs and the active sites of lipase. Moreover, stronger hydrophobic ILs have longer hydrophobic tails (alkyl chains). Recent studies suggest that the hydrophobic tails of the IL cations tend to cluster together and form non-polar domains in the IL-water mixtures with longer tails able to do so more effectively [33,36,37]. These domains are close to the active site entrance of lipase, which may generate a negative impact on the ability of substrates entering or leaving the active sites of lipase and reduce the activity of lipase accordingly [33,36].

Above all, [C_1_C_3_OHim]NTf_2_ can be used in the subsequent experiments as the optimal reaction medium.

### 2.2. Optimization of the Warfarin Yield

**Figure 2:**
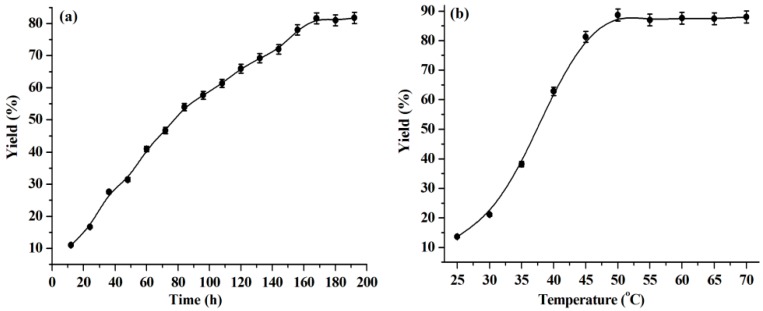
Influence of reaction time (**a**) and temperature (**b**) on the warfarin yield. Reaction conditions: (**a**) BA, 1.0 mmol, water, 0.1 mL, temperature, 45 °C, lipase, 30 mg, 4-HC, 0.5 mmol, [C_1_C_3_OHim]NTf_2_, 2.0 g. (**b**) BA, 1.0 mmol, water, 0.1 mL, reaction time, 168 h, lipase, 30 mg, 4-HC, 0.5 mmol, [C_1_C_3_OHim]NTf_2_, 2.0 g.

Reaction time and temperature are important factors for the warfarin yield. As can be seen from Figure 2a, the warfarin yield keeps increasing in the range of reaction time from 0 to 168 h and remains almost constant above 168 h. Therefore, 168 h is regarded as the optimal reaction time and is used in the subsequent experiments.

The effect of the reaction temperature on the warfarin yield is shown in Figure 2b. The warfarin yield increases with a rise in temperature from 25 °C to 50 °C. It is understood that higher temperature lowers the viscosity of reaction media, which is favorable for reducing the mass transfer resistance and, thus, improves the reaction yield [38,39]. When the reaction temperature is above 50 °C, the warfarin yield keeps constant by further increasing the reaction temperature. Thus, 50 °C is a choice for the optimal reaction temperature.

**Figure 3:**
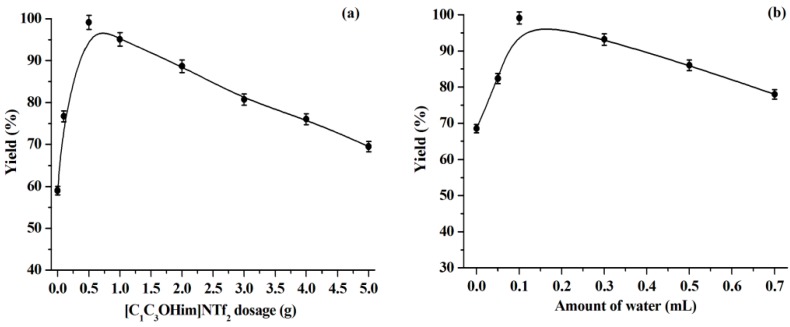
Influence of the dosages of [C_1_C_3_OHim]NTf_2_ (**a**) and water (**b**). Reaction conditions: (**a**) BA, 1.0 mmol, water, 0.1 mL, reaction time, 168 h, temperature, 50 °C, lipase, 30 mg, 4-HC, 0.5 mmol. (**b**) BA, 1.0 mmol, reaction time, 168 ho, temperature, 50 °C, lipase, 30 mg; 4-HC, 0.5 mmol, [C_1_C_3_OHim]NTf_2_, 0.5 g.

Generally, the dosage of solvent has a significant effect on the reaction yield [22,40]. As shown in Figure 3a, the warfarin yield increases with the [C_1_C_3_OHim]NTf_2_ dosage from 0 g to 0.5 g while warfarin yield will decrease with a further increase in the [C_1_C_3_OHim]NTf_2_ dosage. This can be explained by the facts that the addition of the solvent ([C_1_C_3_OHim]NTf_2_) can improve the enzyme stability and activity by maintaining an appropriate microenvironment with mild polarity around the lipase active sites. However, excess solvent can dilute the concentrations of substrates, which will result in a lower reaction yield [22,40]. Since 0.5 g of [C_1_C_3_OHim]NTf_2_ provides the highest warfarin yield (99.1%), it is, thus, selected as the optimal IL dosage in the reactions.

The amount of water plays an important role for the enzymatic catalysis in organic media [22,41]: an essential amount of water can activate the enzyme by increasing the polarity and structural flexibility of the enzyme active sites. Nevertheless, excess water is harmful to the enzyme by facilitating enzyme aggregation, which diminishes the substrate diffusion and eventually leads to the enzyme inactivation. Furthermore, recent studies reported the impact of water dilution on the overall liquid structure and properties of ILs. It is found that the presence of water can reduce the viscosity of ILs and the liquid structure of ILs depends on the IL concentration: there is strong ordering in the local structure of IL at high IL concentrations and a breakdown of the structure at low IL concentrations [15,37]. Therefore, it is necessary to study the impact of the water dosage. The results are shown in Figure 3b. We observed that the addition of an appropriate amount of water can improve the enzyme activity, which achieves the highest warfarin yield (0.1 mL of water), but the warfarin yield decreases with a further increase of the water dosage. Thus, 0.1 mL is regarded as the optimal water dosage.

**Figure 4:**
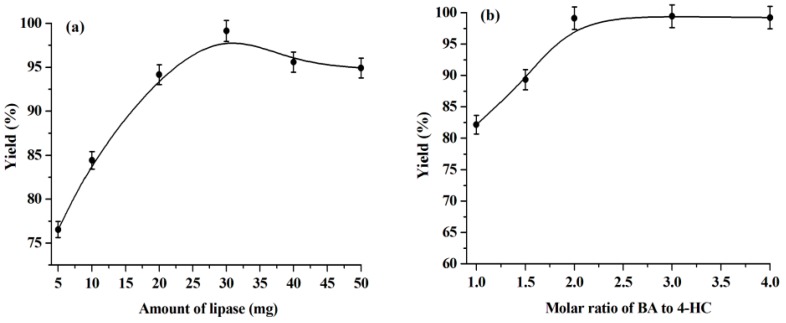
Influence of enzyme dosage (**a**) and molar ratio of BA to 4-HC (**b**). Reaction conditions: (**a**) BA, 1.0 mmol, water, 0.1 mL, temperature, 50 °C, 4-HC, 0.5 mmol, reaction time, 168 h, [C_1_C_3_OHim]NTf_2_, 0.5 g. (**b**) Water, 0.1 mL, temperature, 50 °C, lipase, 30 mg, 4-HC, 0.5 mmol, reaction time, 168 ho, [C_1_C_3_OHim]NTf_2_, 0.5 g.

The effect of enzyme dosage on the warfarin yield has also been researched in this work and the results are shown in Figure 4a. We observed that the warfarin yield increases continuously with the enzyme dosage increasing from 5 mg to 30 mg and decreasing slightly with a further increase of the amount of enzyme. It has been reported that the addition of lipase is essential and higher amounts of lipase have higher activity within a certain range. Beyond that range, the addition of excess lipase would no longer enhance the warfarin yield since an optimal amount of water is required to activate the added enzyme. In this way, at a non-optimal water dosage, some portion of the enzyme may be inactive [22,42]. In light of this, 30 mg of lipase is selected for the following experiments.

Figure 4b illustrates the effect of the molar ratio of BA to 4-HC on the warfarin yield. The highest yield is obtained at a 2:1 molar ratio of BA to 4-HC. Further increasing the number of equivalents of BA does not improve the warfarin yield. Therefore, 2:1 is taken as the optimal molar ratio of BA to 4-HC.

### 2.3. Reusability of Lipase and [C_1_C_3_OHim]NTf_2_

**Figure 5:**
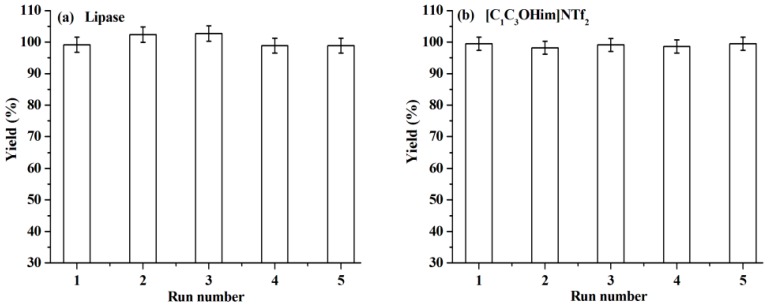
Reusability study of lipase (**a**) and [C_1_C_3_OHim]NTf_2_ (**b**) under the optimal reaction conditions.

**Figure 6:**
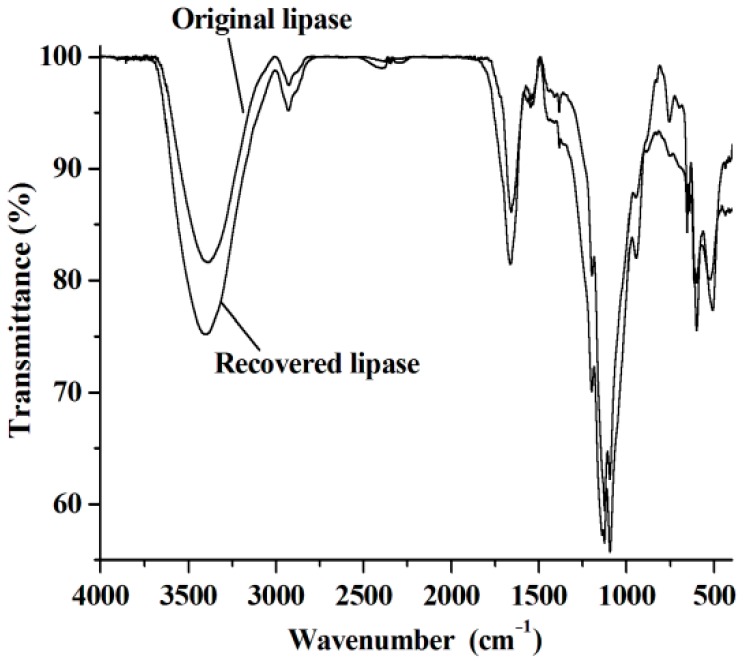
The FT-IR spectra of the original lipase and the recovered one.

The reuse of lipase and [C_1_C_3_OHim]NTf_2_ is clearly preferable from both environmental and economic points of view. To reuse lipase, the reaction mixture is dissolved with acetone after the end of the reaction. After centrifugation, the upper phase (acetone phase) is collected to recover [C_1_C_3_OHim]NTf_2_ and warfarin. The bottom phase is lipase, which is washed with acetone several times until no [C_1_C_3_OHim]NTf_2_, BA, 4-HC, and warfarin are detected in the washings. After being dried at 25 °C for 24 h, the recovered lipase is loaded into the reaction mixture for a new reaction cycle under the optimal reaction conditions. The catalytic performance of lipase after recycling is shown in Figure 5a. As can be seen, no obvious changes in warfarin yield are observed after five consecutive cycles, which exhibits excellent reusability of lipase. The FT-IR spectra of lipase before and after use were measured and the results shown in Figure 6 indicate that the FT-IR spectrum of the recovered lipase is identical to that of the fresh one, which suggests that lipase remains its native conformation after reaction. This explains why the lipase can be reused without the loss of its catalytic performance.

To reuse [C_1_C_3_OHim]NTf_2_, the acetone phase, which is obtained from the recycling of lipase, is distilled to remove acetone. The residue is mixed with NaOH solution (the molar amount of NaOH is equal to the theoretical amount of warfarin), the upper phase (NaOH solution) of the resultant mixture is collected to isolate warfarin, and the bottom phase is [C_1_C_3_OHim]NTf_2_, which contains 5.5% of BA, 2.0% of 4-HC, and 1.2% of warfarin. After being dried at 60 °C for 24 h, the recovered [C_1_C_3_OHim]NTf_2_ can be used for the next reaction cycle. Figure 5b shows that the warfarin yield stays almost constant after five runs, which suggests that [C_1_C_3_OHim]NTf_2_ also possesses excellent reusability.

### 2.4. Separation of Warfarin from the Reaction Mixture

As mentioned above, after the end of the reaction, acetone is used to dissolve the product mixture and the resultant acetone phase is distilled to remove acetone. The resultant residue is mixed with NaOH solution to generate the aqueous phase containing warfarin sodium. In doing so, the product can be separated from the reaction mixture. Then, the pH of the aqueous phase is adjusted to about 3.0 with acetic acid and a light yellow precipitate is obtained (crude warfarin). The resultant crude warfarin is washed with the mixture of ethanol and acetic acid solution (pH 2.5) (3:5, *v*/*v*) several times until white powder is obtained. Part of warfarin is lost during washings and the final warfarin yield is 83.7% and the purity of warfarin (determined via the HPLC method) is 99.3%. The specific rotation of the synthesized warfarin is −1.8°. It is known that the specific rotations of *S*-warfarin and *R*-warfarin are −25.5 ± 1° and +24.8 ± 1°, respectively [43]. Therefore, the synthesized warfarin is a racemic mixture, which is in agreement with the clinically available warfarin [44].

## 3. Materials and Methods

### 3.1. Reagents

Lipase from *Candida ruosa* (type VII, activity, ≥700 unit mg^–1^) was purchased from Sigma-Aldrich Co. (St. Louis, MO, USA). 4-Hydroxycoumarin (4-HC, 98%), warfarin sodium (98%), and benzylideneacetone (BA, ≥99%) were obtained from Aladdin Reagent Co. (Shanghai, China). *n*-Octanol (98%), sodium 1-heptanesulfonate (99%), and acetonitrile (99.9%) were obtained from Energy Chemical Co. (Shanghai, China). The ILs, [C_1_C_4_im]BF_4_ (99%), [C_1_C_4_im]PF_6_ (99%), and [C_1_C_4_im]NTf_2_ (99%) were supplied by the Lanzhou Institute of Chemical Physics, Chinese Academy of Sciences (Lanzhou, China). The ILs, [C_1_C_6_OHim]NTf_2_, [C_1_C_3_OHim]NTf_2_, [C_4_C_3_OHim]NTf_2_, ([C_4_C_11_OHim]NTf_2_, and [C_1_C_11_OHim]NTf_2_ were synthesized on the basis of our early work [45]. All the other reagents were of an analytical grade unless stated otherwise. Ultrapure water (18.2 MΩ cm) used throughout the experiments was produced by an Aquapro purification system (Aquapro International Co., Ltd., Dover, DE, USA).

### 3.2. Determination of Warfarin

To monitor the reaction, the ultraviolet-visible (UV-Vis) spectra of the raw materials and the products were measured by a TU-1810 spectrophotometer (Purkinje General Instrument Co., Beijing, China) (Figure S1). As can be seen from the spectra of the reaction mixture (Figure S1), after reaction, the absorbance of BA decreases remarkably and the absorption peaks of warfarin appear, which suggests that the reaction occurred. To more accurately measure the concentrations of warfarin in the reaction media, an Agilent 1200 high performance liquid chromatograph (HPLC, Santa Clara, CA, USA) equipped with a variable wavelength detector (VWD) and an auto-sampler was used. An Amethyst C18-H column (4.6 mm × 150 mm, 5 μm, Sepax Technologies Inc., Newark, NJ, USA) was used to separate warfarin. The mobile phase was the mixture of acetonitrile and 0.10% (*v*/*v*) acetic acid aqueous solution (55:45, *v*/*v*), the flow rate was 0.70 mL min^−1^, the injection volume was 5.0 μL, the column temperature was 30 °C, and the detection wavelength was set at 308 nm.

### 3.3. Determination of the Hydrophobic Parameters of the ILs

The *n*-octanol–water partition coefficients (*P*_ow_), which are usually expressed as log*P*_ow_ values, are regarded as measures of hydrophobicity of organic substances. The log*P*_ow_ values of the ILs used in this work were measured according to the reported methods [46,47]: the IL solutions (5.0 × 10^–4^ mol L^–1^ for each) and the *n*-octanol were presaturated with *n*-octanol and water, respectively. As an example, the mixture composed of IL solution (10.0 mL) and *n*-octanol (10.0 mL) was stirred for 30 min at 25 °C. Then the *n*-octanol and the water phases were separated by centrifugation. The concentrations of the ILs both in the *n*-octanol and water phases were determined by the HPLC method. The chromatographic conditions were listed as follows: injection volume, 2.0 μL, flow rate, 0.80 mL min^–1^, detection wavelength, 220 nm, mobile phase, a mixture of acetonitrile and 2.0 × 10^–3^ mol L^–1^ of aqueous sodium 1-heptanesulfonate solution (50% (*v*/*v*) of acetonitrile for determining [C_4_C_11_OHim]NTf_2_ and [C_1_C_11_OHim]NTf_2_; 40% (*v*/*v*) of acetonitrile for measuring the remaining ILs).

The *P*_ow_ value is calculated according to the equation below [46,47].

(1) $$P_{\mathrm{ow}}=\frac{C_{n-\mathrm{octanol}}}{C_{\mathrm{water}}}$$

In the equation, *C_n_*_-octanol_ and *C*_water_ are the concentrations of a given IL in the *n*-octanol and water phases, respectively. The log*P*_ow_ value of DMSO is from the reported literature [48].

### 3.4. Synthesis of Warfarin

Typically, a mixture composed of 4-HC (0.5 mmol), BA (1.0 mmol), IL (0.5 g), lipase (30 mg), and water (0.1 mL) was kept stirring for 168 h at 50 °C. After the reaction, the concentration of warfarin in the prepared product was measured by the HPLC method. The product pre-processing procedure was as follows: at the end of the reaction, the product mixture was diluted to 20 mL with ethanol, filtrated to remove lipase, and then the resulting ethanol solution was further diluted 10 times with ethanol before HPLC measurements. The percent yield is defined as the ratio of the actual amount of warfarin to the theoretical amount.

### 3.5. Measurements of Fourier Transform Infrared (FT-IR) Spectra

The FT-IR spectra were measured by the KBr pressed disc method on a Bruker V70 FT-IR spectrophotometer (Bruker Optic GmbH, Ettlingen, Germany).

### 3.6. Measurements of the Specific Rotation of the Synthesized Warfarin

The measurements of the specific rotation of the synthesized warfarin were performed on a SGW-1 automatic polarimeter (Shanghai instrument physical optics instrument Co., Ltd., Shanghai, China). The temperature was 25 °C and the concentration of warfarin was 25 g L^–1^ (solvent, ethanol).

All the above experiments were conducted in triplicate and the data presented in this work are averages of the obtained values.

## 4. Conclusions

In this work, a series of ILs were used as the reaction media of the lipase-catalyzed Michael addition of 4-HC to BA. Compared with the conventional solvent DMSO and the dialkylimidazolium-based ILs, the hydroxyl-functionalized IL [C_1_C_3_OHim]NTf_2_ is a more ideal reaction medium because the hydroxyl group in the IL greatly promotes the lipase activity due to its H_2_O-mimicking property and hydrogen-bonding functionality. The lipase activity decreases with an increasing alkyl chain length of the IL cation, which can be ascribed to the direct interactions of the IL cations with the active sites of lipase and the non-polar domain formation of the IL cations at the active site entrance of lipase. After the end of the reaction, lipase and [C_1_C_3_OHim]NTf_2_ could be recovered and reused no less than five times without a decrease of the catalytic performance of lipase.

## Figures and Tables

**Table 1:** Names and abbreviations of the ILs used in this work.

Name	Abbreviation
1-Butyl-3-methylimidazolium tetrafluoroborate	[C_1_C_4_im]BF_4_
1-Butyl-3-methylimidazolium hexafluorophosphate	[C_1_C_4_im]PF_6_
1-Butyl-3-methylimidazolium bis(trifluoromethylsulfonyl)imide	[C_1_C_4_im]NTf_2_
1-Methyl-3-(6-hydroxyhexyl) imidazolium bis(trifluoromethylsulfonyl)imide	[C_1_C_6_OHim]NTf_2_
1-Butyl-3-(3-hydroxypropyl) imidazolium bis(trifluoromethylsulfonyl)imide	[C_4_C_3_OHim]NTf_2_
1-Methyl-3-(3-hydroxypropyl) imidazolium bis(trifluoromethylsulfonyl)imide	[C_1_C_3_OHim]NTf_2_
1-Butyl-3-(11-hydroxyundecyl) imidazolium bis(trifluoromethylsulfonyl)imide	[C_4_C_11_OHim]NTf_2_
1-Methyl-3-(11-hydroxyundecyl) imidazolium bis(trifluoromethylsulfonyl)imide	[C_1_C_11_OHim]NTf_2_

## Supplementary Materials

The following are available online, Figure S1: UV-Vis absorption spectra of warfarin, 4-HC, BA, and the reaction mixture before and after the reaction.
