# Supplementary material for: Ionic Liquids: Efficient Media for the Lipase-Catalyzed Michael Addition
Source: Molecules. 2018 Aug 27;23(9):2154. doi: 10.3390/molecules23092154 (PMC6225191; doi:10.3390/molecules23092154)
Supplement: Supplementary file 1 [file molecules-23-02154-s001.pdf]

Supplemental Information

Ionic Liquids: Efficient Media for the Lipase-Catalyzed Michael Addition

Yunchang Fan*, Dongxu Cai, Xin Wang and Lei Yang

College of Chemistry and Chemical Engineering, Henan Polytechnic University, Jiaozuo 454003, China; CDX940407@163.com (D.C.); asnol21@163.com (X.W.); xcy78413@tom.com (L.Y.)

*Correspondence: fanyunchang@hpu.edu.cn; Tel.: +86-391-398-6813

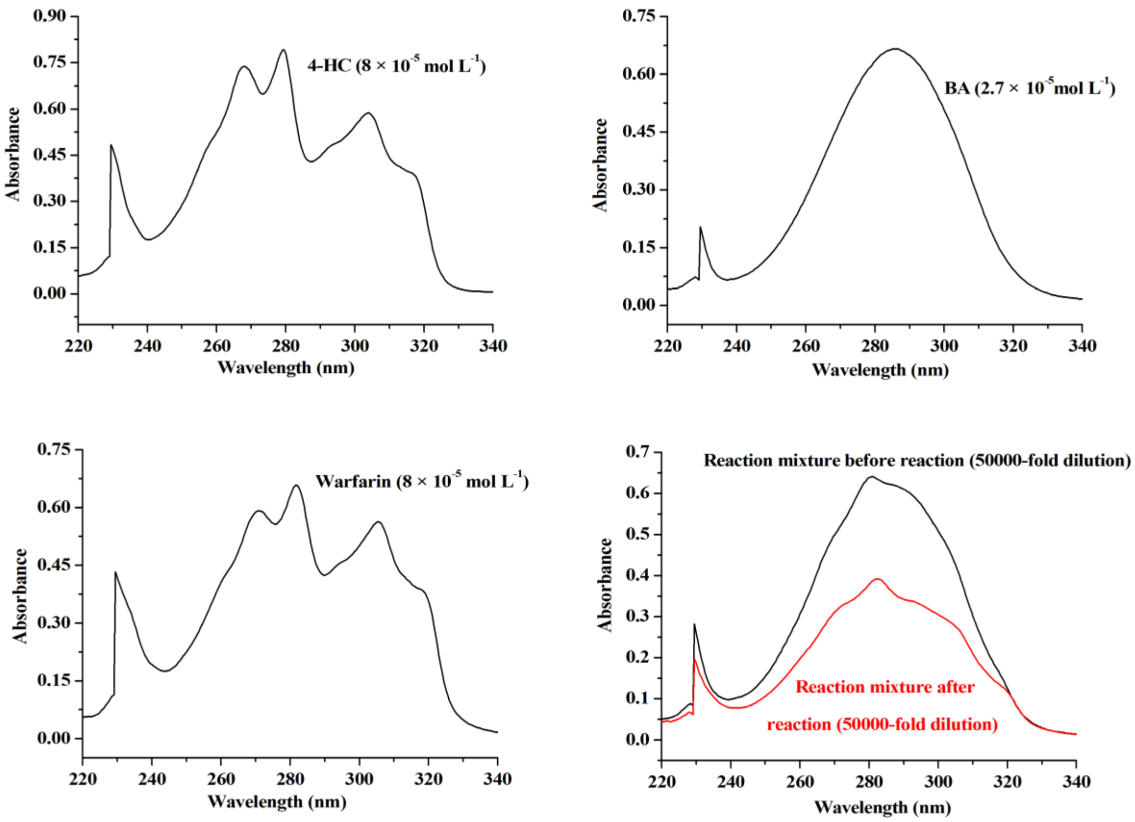

Figure S1. UV-Vis absorption spectra of warfarin, 4-HC, BA and the reaction mixture before and after reaction.
